# Magnetorheological Fluid Utilized for Online Rotor Balancing

**DOI:** 10.3390/mi16101083

**Published:** 2025-09-25

**Authors:** Valentin Schreiner, Jürgen Maas

**Affiliations:** Mechatronic Systems Laboratory, Technical University of Berlin, 10623 Berlin, Germany

**Keywords:** magnetorheological fluid, vibration, online rotor balancing, active balancing, multiphysics modeling, non-Newtonian fluid

## Abstract

Unbalance in rotating machinery causes significant vibrations, reducing lifespan and efficiency. This study overcomes the limitations of conventional offline balancing by introducing an online rotor balancing system utilizing Magnetorheological Fluid (MRF). The system employs three chambers containing MRF and balancing spheres. A hybrid magnetic system is designed, combining permanent magnets for fixing in the balanced state and a stationary electromagnet for contactless control of the sphere position during balancing. A control algorithm was developed based on multiphysics modeling and experimental parameterization. Experimental validation demonstrated the high effectiveness of reducing initial rotor unbalance, thereby confirming the feasibility of the proposed concept. The hybrid magnetic design provides inherent multistability and energy efficiency, making it a promising and effective solution for active vibration reduction in rotating machinery.

## 1. Introduction

Rotating machinery is fundamental to countless industrial applications, while its operational capability, lifespan, and safety are critically dependent on maintaining a high degree of rotational balance. Rotor unbalance, due to manufacturing tolerances, material inhomogeneities, or in-service changes like wear, induces significant vibrations that can lead to increased noise, accelerated component wear, reduced precision, or even catastrophic mechanical failure. Conventional balancing methods typically involve offline procedures where the machine is stopped, the unbalance is analyzed, and corrective masses are manually added or removed [1]. Although these methods are effective for static or initial unbalance, they cannot compensate for changes that occur during operation. This has motivated the development of online or active balancing systems, detecting and correcting unbalance in real-time and during operation.

Based on the underlying control system, these online balancing systems can be divided into passive and active systems. Passive systems utilize the vibrations at supercritical speed to correct the unbalance and do not require measurement of the unbalance condition. One limitation of these concepts, which are often designed as ball balancers [2,3,4] or liquid balancers [5,6], is the amplification of the vibrations in the subcritical range. In [7], the authors reduced this effect by a speed-dependent release of the balancing balls. Ref. [8] presents a combination of several automatic ball balancers that are spatially decoupled from the rotor shaft. Equipped with its own drives, this concept is positioned between the active and passive systems. In contrast, active systems require identifying the unbalance and executing corrective measures. This process requires transferring energy, information, or materials to the rotating system [9,10]. The active balancing system presented in this work overcomes this challenge using magnetorheological fluid (MRF) and enables a multistable behavior based on a hybrid magnet system. The controlling magnetic energy required for balancing is transmitted without contact via an air gap.

MRF are smart materials that exhibit a rapid change in their rheological properties, specifically their yield stress, when an external magnetic field is applied [11,12]. Apart from the balancing application presented here, MRF is mainly used for dampers [13,14] with variable characteristic curves as well as brakes and clutches [15]. The application of MRF for balancing has been investigated in prior work. However, these previously presented systems are characterized by a complex design and a lack of multistability, thus requiring a continuous energy input to maintain a corrective state [16,17]. The authors have previously proposed a multistable system in [18]. The principle of MRF displacement employed in that system, however, was proved difficult to control. A subsequent approach, explored in [19], is based on inducing concentration shifts within the fluid.

The following work builds on the concept presented in [20]. This study served only to demonstrate the functionality of the system, but did not contain any in-depth approaches for modeling and optimizing the system’s behavior. The shear forces were investigated only for the static case without the intervention of the electromagnet using a two-dimensional simulation of the magnet system. The presented balancing run used an iterative controller that required 19 discrete interventions for balancing in this first version. To overcome this limitation, this article presents an in-depth modeling approach based on a comprehensive three-dimensional multiphysics model and a time-efficient control algorithm with high control quality. The methodology developed is derived, described, and experimentally validated in detail in this article. The concept of the online balancing system and the underlying functional principle are introduced in detail in Section 2, followed by an elaborate analysis of the hybrid magnetic circuit utilized for the balancing in Section 3. Section 4 contains the development of a comprehensive multiphysics model in three-dimensional space, considering the non-Newtonian behavior of the MRF. The new approach includes pressure forces and models the dynamic movement of the balancing sphere under the influence of the controlling magnetic field. Section 5 presents the system for experimental validation as well as the parameterization process, which is used to develop the new time-efficient balancing algorithm introduced in Section 6. Finally, Section 7 demonstrates the behavior of the overall system using an experimental balancing run. In this run, only two interventions are required to reach the balanced state, confirming the ability to efficiently reduce rotor unbalance during operation.

## 2. Functional Principle and Design of the Online Balancing System

Section 2 outlines the concept and functional principle of the balancing system. Specifically, we discuss how balancing spheres are used to shift the center of mass and lock in a balanced state to counteract vibrations. We also explain how gravity is used to reset the system for its next operation.

### Principle of Mass Compensation in a Three-Chamber System and Its Limitation

**Figure 1:**
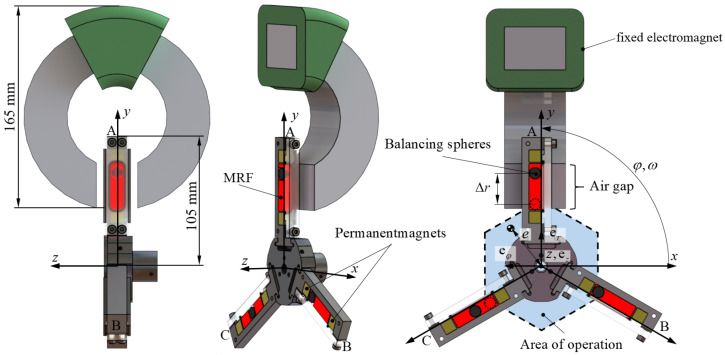
Design of the balancing head with three chambers offset by 120°, MRF and balancing weights displayed for $\varphi =90^{\circ }$. The displacement of the balancing weights in chambers A and B results in an eccentricity *e* of the center of mass, which is shown enlarged in the illustration.

The principle of operation involves three balancing chambers mounted in a star-shaped configuration offset by 120 degrees on the rotor, as illustrated in Figure 1. Each chamber contains MRF, permanent magnets, and a balancing sphere. The selection of the balancing mass is crucial, as the materials must be selected based on their density in relation to the MRF. For instance, high-density tungsten or steel spheres may be used, as well as low-density masses like polymer or foam spheres.

Without the permanent magnets in each chamber, centrifugal forces would cause a radial displacement of the balancing masses. While a high-density sphere would drift towards the outer radius of the chamber, a low-density sphere is pushed by the denser MRF towards the axis of rotation. In both scenarios, the movement of the mass relative to the fluid changes the mass distribution and therefore has the potential for correcting the rotor unbalance. Since balancing requires controlled movement of the spheres, a hybrid system was developed that combines permanent magnets on the rotor with a stationary electromagnet.

In contrast to passive balancing systems, this approach enables active control over the position of the balancing masses. This control is achieved by influencing the rheological state of the MRF via a magnetic field, which is manipulated by the external electromagnet shown in Figure 1. The formation of the yield point $\tau_{0}\left(B\right)$ under the influence of the permanent magnetic field, “freezes” the positions of the balancing mass within the MRF. By precisely timing the activation and deactivation of the electromagnet, the balancing masses can be fixed at optimal locations to achieve a balanced state. This capability creates a multistable system, as the balancing spheres remain permanently in the desired radial position within their chambers. A key advantage of this design is its inherent stability. Once the optimal positions are locked, the balanced state is maintained. This balancing configuration persists even if the machine is stopped and restarted, as the solidified MRF holds the spheres firmly in place, ensuring the system remains balanced without requiring a new cycle for each operation. Due to the remanent magnetic field of the permanent magnets, the structure presented can also counteract the tendency of the MRF to segregate under high rotational and gravitational acceleration as shown in [19,21].

Several key operational characteristics of the system can be derived based on the functional principle discussed. A fundamental constraint of the design is that during rotation, centrifugal force only permits the balancing masses to move radially outward. Consequently, at the start of any balancing procedure, all spheres must be located at their innermost position, closest to the center of rotation. Despite this limitation, the arrangement of three chambers offset by 120 degrees allows the system to compensate for any balance vector within its working plane. This is achieved by precisely displacing the appropriate two of the three masses outward to create a resultant correction vector of the required magnitude and direction. This capability defines the system’s operational workspace, which takes the form of a hexagon, as schematically highlighted in the blue overlay of Figure 1. The inner radius of the hexagon can be determined by calculating the maximum possible mass eccentricity *e* caused by the movement of one balancing sphere. This parameter is the product of the displacement from the initial position, Δr as depicted for Chamber A of Figure 1 and the mass difference between the sphere and the displaced MRF. Equation (1) models this relationship. Herein, $m_{\mathrm{rotor}}$ represents the total mass of the entire rotor.

(1) $$e=\frac{V_{\mathrm{sphere}}\;\cdot \;(\rho_{\mathrm{sphere}}-\rho_{\mathrm{MRF}})}{m_{\mathrm{rotor}}}\;\cdot \;\Delta r.$$

Important characteristic data of the design analyzed in this work are shown in Table 1.

After a balancing process is completed, the spheres are fixed in their compensation positions and are therefore no longer at their initial starting point. To begin a new balancing cycle, the system must first be reset to this initial state. This reset procedure is accomplished by leveraging Earth’s gravitational field. To do so, each chamber is sequentially rotated into an upright, vertical position within the air gap of the electromagnet. By activating the electromagnet for a specific chamber, the MRF is returned to its liquid state. Under the influence of gravity, the balancing sphere then moves back to its starting position near the center of the rotor. This process is repeated for each chamber, ensuring the system is ready for the next balancing operation.

## 3. Development and Analysis of the Magnetic Excitation System

Section 3 presents the hybrid magnetic system containing the permanent magnet excitation on the rotor, as well as the electromagnetic control system, and analyzes their interaction.

### 3.1. Permanent Magnet Circuit in the Chamber for Stabilizing the Balancing Mass

**Figure 2:**
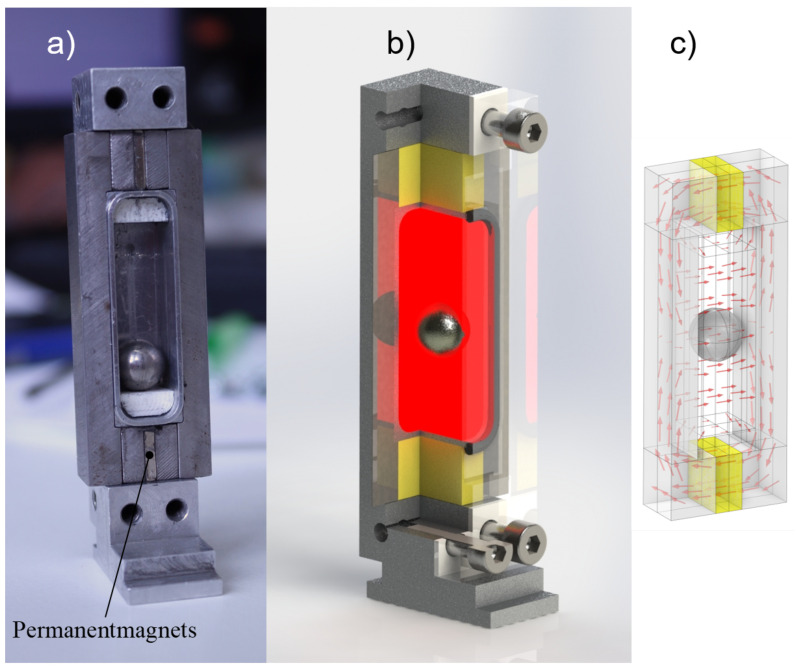
Structure of the balancing chamber. (**a**) Open chamber without MRF, (**b**) sectional view of the CAD model, (**c**) schematic view of the magnetic flux due to the permanent magnets.

The permanent magnetic system provides an initial state in which the MRF is exposed to a homogeneous magnetic field. The role of this remanent field is to ensure a stable state and to position the balancing sphere at a precise location with the assistance of the magnetically controlled yield point $\tau_{0}\left(B\right)$. Depending on the intended maximum speed, a minimum magnetic flux density must be achieved in the chambers. At the same time, the permanent magnetic system must be designed in such a way that a weakening of the field by the electromagnet is possible in order to control the displacement of the balancing spheres. The fundamental design of the chambers utilized and the magnetic circuit employed are illustrated in Figure 2.

The source of the remanent magnetic flux is provided by the permanent magnets, which are mounted in oppositely polarized pairs on the inner and outer walls of the chamber and are adjacent to the flux-carrying components (11SMn30), while two flux-guiding bars close the circuit via the permanent magnets. Due to the opposing polarization, the magnetic field is conducted through the MRF inside the chamber. The resulting path of the magnetic flux is illustrated qualitatively in Figure 2b.

### 3.2. Stationary Electromagnet and Interaction with the Rotating Chamber System

**Figure 3:**
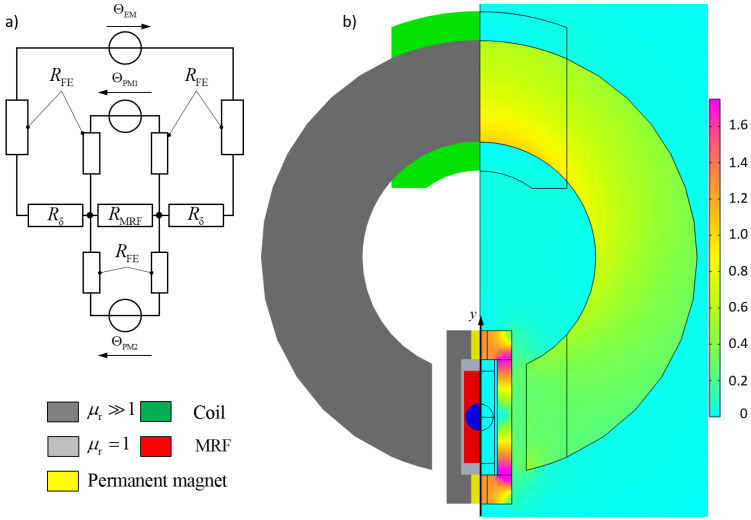
(**a**) Corresponding electrical circuit diagram of the hybrid magnetic circuit, (**b**) sectional view illustrating one of the chambers located in the air gap of the electromagnet, color scheme: magnitude of the magnetic flux density |B| in T.

The purpose of the stationary electromagnet is to enable precise control over the field distribution across all three chambers during the rotation process. Due to the high level of dynamics required, a core composed of electrical steel sheets is used to prevent eddy currents. The core is fitted with a surrounding winding of N=400. Figure 3 presents the schematic structure of the system and a simulation of the magnetic flux density with an active electromagnet during the phase in which the chamber passes through the air gap of the electromagnet. Figure 3 presents an illustration of the corresponding electrical circuit diagram. Herein $\Theta_{\mathrm{PM1}}$ and $\Theta_{\mathrm{PM2}}$ describe the magnetic voltages of the two permanent magnets, while $\Theta_{\mathrm{EM}}$ symbolizes the additionally activated electromagnet. The resistances in the circuit diagram symbolize the reluctance of the magnetic circuit and are of different magnitudes. While $R_{\mathrm{Fe}}$ describes the reluctance of the flux-carrying material in the electromagnet and chamber, which have negligible values, $R_{\delta }$ modeling the air gaps between the chamber and the electromagnet, and $R_{\mathrm{MRF}}$ considering the magnetic resistance of the MRF itself, representing significantly larger values.

**Figure 4:**
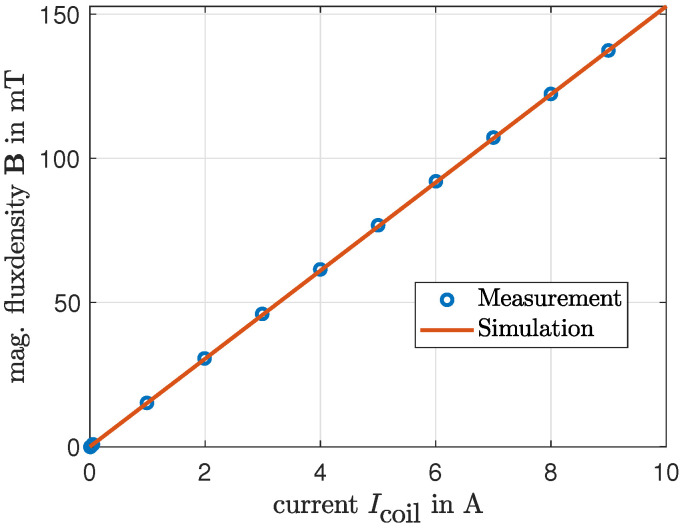
Comparison between measurement and simulation of the current-flux density characteristic of the electromagnetic excitation system measured in the air gap without the chamber or MRF present.

To analyze the displacement of the balancing body in Section 4, precise information on the magnetic flux density within the MRF is required. To determine this quantity, the magnetic system is modeled in COMSOL Multiphysics 6.3. Due to the various geometric states resulting from the rotation of the chamber, a three-dimensional simulation is necessary for this investigation. For validation of the model, measurements of the magnetic flux density are conducted using the experimental setup described in Section 5, and the results are compared with the simulation. A Teslameter (model AS-NTP 0.6, from the company Projekt Elektronik Mess- und Regelungstechnik GmbH, Berlin, Germany) is used for these measurements. Figure 4 shows the results of a measurement conducted on the isolated electromagnetic excitation system without the chamber or MRF present. In this measurement, the magnetic flux density was recorded at the center of the air gap and is compared with the simulation results of the isolated electromagnetic excitation system. The characteristic curve of the electromagnet exhibits linear behavior in the investigated range and is accurately reproduced by the simulation.

**Figure 5:**
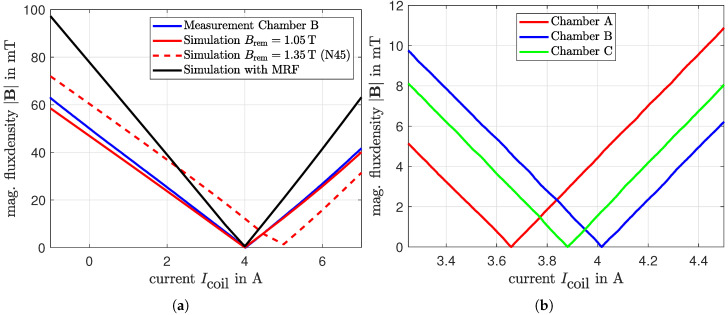
Analysis of the current-flux density characteristic of the hybrid magnetic system. (**a**) Comparison between measurement in chamber B and simulation is used to adjust the remanent flux density in the simulation. (**b**) Comparison between the three chambers shows a significant shift in the compensation current.

Since a non-intrusive and non-interfering measurement within the MRF, when using a Teslameter, is not feasible, the behavior of the permanent magnet system and its interaction within the air gap are investigated using an empty chamber, without the MRF or the balancing body. The results for Chamber B are presented in Figure 5a. Compared to the measurements, the simulation result using the manufacturer-specified magnetization exhibits a significant parallel shift with regard to current values. Therefore, to correct the simulation, the remanent flux density $B_{\mathrm{rem}}$ of the permanent magnets is iteratively reduced until it matches the experimental data at the compensation point $I_{\mathrm{coil}}=4\;A$. Using this adjusted remanent flux density, the simulation is then expanded to include the MRF and the balancing body. The resulting field distribution with the MRF-filled chamber is also depicted in Figure 5a. Figure 5b illustrates the compensation point of the three chambers relevant for the sphere movement. With regard to the point with minimum field (compensation point), significant deviations in the current to be set are apparent, which is presumably due to tolerances in the production of the permanent magnets, while the quantitative characteristics beyond this point correspond well. Table 2 contains the numerical values of the corresponding currents and the offset ΔI of the compensation point. Chamber B is used as the reference value in Table 2 and the subsequent analyses due to the adapted simulation.

**Figure 6:**
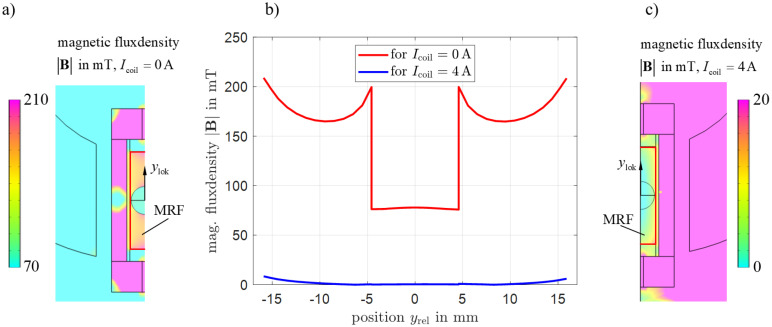
Distribution of magnetic flux density within the chambers. (**a**) Distribution with deactivated electromagnet, (**b**) magnetic flux density profile along the center axis of the chamber, (**c**) distribution with maximum field displacement.

In order to analyze the homogeneity of the magnetic field inside the chamber, Figure 6 shows the magnetic flux density for two extreme cases: deactivated electromagnet yellowFigure 6a and under applied compensation current yellowFigure 6c at the center plane (z=0). The area in which the MRF is located is enclosed by a red boundary line. yellowFigure 6b shows the magnetic flux density along the center axis of the chamber. When the electromagnet is deactivated, the MRF reaches values between 208 mT and 165 mT. Significantly lower values occur inside the tungsten carbide sphere in the range $y_{\mathrm{lok}}=\pm 4.5\;\mathrm{mm}$. In the case of compensation (I = 4 A), values of up to 8 mT are obtained at the sides, while in the center, the magnetic field is almost completely displaced (B < 1 mT).

**Figure 7:**
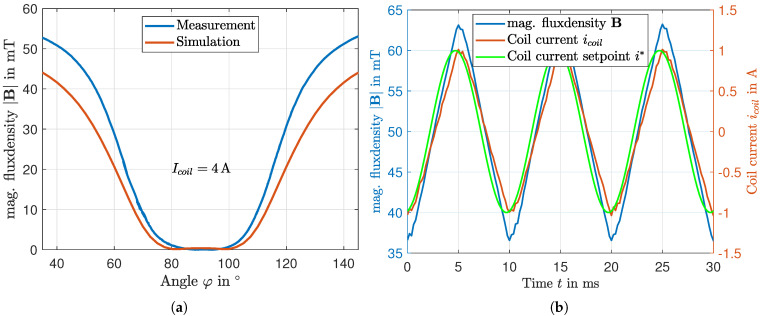
(**a**) Magnetic flux density as a function of the angular position passing the active air gap. (**b**) Investigation of the dynamic behavior of the hybrid magnetic circuit. No phase shift can be identified between the measured values of current and magnetic flux density.

For the simulation in Section 4, the magnetic field strength must be defined as a function of the corresponding angle at each time $\varphi \left(t\right)$. Consequently, Figure 7a shows the magnetic flux density as a function of angular position during one rotation through the air gap. Similar to the previous figures, the measured data are compared with the corresponding simulation results. The results show a very good match at the center of the air gap at approximately φ=90°, where the flux density reaches its minimum. However, as the chamber is displaced from this central position, the deviations between the measurement and simulation increase. One possible reason for this difference is the adjustment of the remanent flux density mentioned earlier. The magnetic circuit was optimized specifically for the compensation point through simulations, which leads to inaccuracies when properties deviate from this point.

Due to geometric and technical constraints, measuring the magnetic flux density at the actual operating speed is not feasible. In order to verify the validity of the statically measured characteristic curve for dynamic operation, the dynamics of the hybrid magnetic circuit are investigated instead. For this purpose, a periodic current with a frequency of $f_{\mathrm{Icoil}}\;=\;100\;\mathrm{Hz}$ is applied, and both the resulting coil current $I_{\mathrm{coil}}$ and magnetic flux density B are measured. The results are presented in Figure 7b.

The diagram reveals a certain phase shift between the setpoint current $I_{\mathrm{coil}}^{*}$ and the measured coil current $I_{\mathrm{coil}}$. In contrast, a not detectable phase shift is observed between the measured coil current $I_{\mathrm{coil}}$ and the resulting magnetic flux density B, as the two curves are in phase. From this observation, it can be concluded that the electrical subsystem (the current control loop) exhibits an inherent small system delay, whereas the magnetic domain reacts almost instantly to the current. This finding is crucial, as it supports the conclusion that the profile of the magnetic flux density as a function of angle is independent of rotational speed and enables a statically determined profile to derive the magnetic field-dependent fluid properties as a basis for solving the Navier-Stokes-Equation in Section 4.

**Figure 8:**
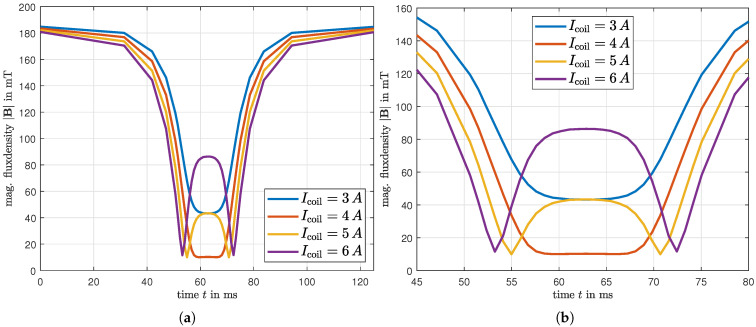
Absolute value of the magnetic flux density over time, average over the fluid volume of the chamber, (**a**) during a complete rotation, (**b**) passing through the air gap.

Figure 8 shows the simulation result of the average magnetic flux density B within the MRF-filled chamber in configuration 2, containing the balancing sphere. The plot illustrates the time course for a constant angular velocity of ω= 50 rad/s at different coil currents $I_{\mathrm{coil}}$. The x-axis in Figure 8a corresponds to the passing of the active air gap during one complete revolution. In Figure 8b the same results are shown magnified for the region of the air gap. These position- and current-dependent characteristics of the magnetic flux density in the MRF serve as direct inputs for the subsequent analysis of the balancing sphere’s displacement using the Navier-Stokes-Equation, as detailed in Section 4. If the current is increased above the compensation value of 4A, the magnetic flux in the relevant air gap increases, which leads to an increase in the yield strength of the MRF and restricts the movement of the spheres.

## 4. Modeling the Non-Newtonian Fluid Behavior and the Sphere Dynamic

The rotor balancing procedure introduced in Section 2 enables the compensation of any unbalance within the system’s operating range via the targeted displacement of two balancing masses. Accordingly, the aim of Section 4 is to model the behavior of these balancing masses in a chamber-fixed reference frame using COMSOL Multiphysics. This provides insight into the chamber’s interior, which is experimentally difficult to observe. Initially, the rheological behavior of the MRF is modeled. Next, the forces acting on the balancing mass are described and used to derive its equation of motion. The described physical relationships, combined with the magnetic domain, are then implemented and numerically analyzed in COMSOL Multiphysics. Beyond a deeper understanding of the system’s behavior, the developed equations can also be utilized for the design and performance evaluation of future systems.

### 4.1. Modeling of the Non-Newtonian Fluid Behavior of the MRF Used

**Figure 9:**
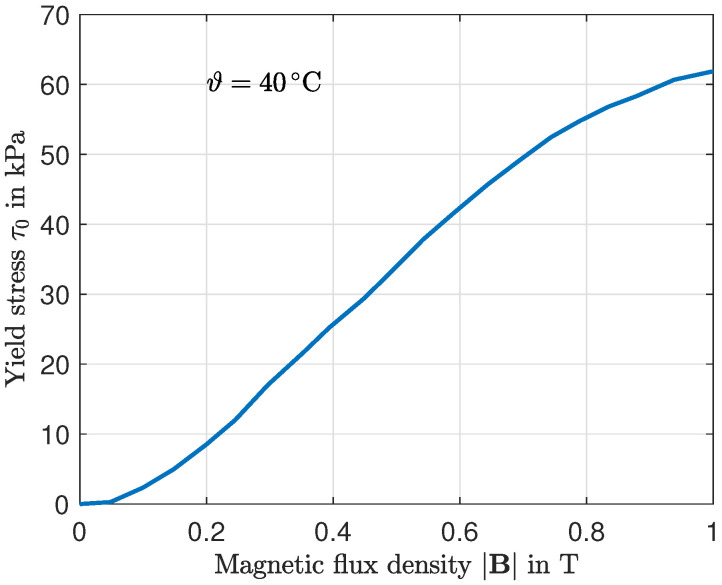
On-state behavior of the MRF Basonetic 5030. Development of yield stress under the influence of a magnetic field [22].

The MRF used in this study is Basonetic 5030, manufactured by BASF (Ludwigshafen, Germany). Relevant technical specifications of the fluid are summarized in Table 3. The crucial characteristic of the MRF in the context of the present work is the formation of a yield stress $\tau_{0}$ when exposed to an external magnetic field, allowing the control of the shear stress. The yield stress characteristic versus the magnetic flux density is shown in Figure 9.

The flow conditions within the chamber, described by the Navier-Stokes-Equations, are the decisive factor for the movement of the balancing sphere. The modeling in COMSOL Multiphysics is based on the assumption of an incompressible flow with isotropic material properties and is given by:

(2) $$\rho \frac{\partial u}{\partial t}+\rho \left(u\;\cdot \;\nabla \right)u=\nabla \left[-pI+K\right].$$

Here, u denotes the velocity field, ρ the mass density, and *p* the pressure. I is the identity tensor, and K is the viscous shear stress tensor, which accounts for the internal shear stresses, given by:

(3) $$K=\eta \left(\nabla u+{\left(\nabla u\right)}^{T}\right).$$

The Bingham-Papanastasiou-Model [23] has proven effective in describing the material behavior of MRF in the past [18]. This model considers the dependence of the shear stress on the magnetic flux density B, while neglecting its dependence on shear rate $\dot{\gamma }$.

(4) $$\tau =\eta \;\cdot \;\dot{\gamma }+\tau_{0}\left(|B|\right)\;\cdot \;\left[1-e^{-m_{p}\;\cdot \;\left|\dot{\gamma }\right|}\right]\;\cdot \;\mathrm{sgn}\left(\dot{\gamma }\right).$$

For integration into the Navier-Stokes-Equation, the internal shear stress can be divided by the shear rate and thus converted into the apparent viscosity $\eta_{\mathrm{app}}$ as shown in Equation (5). Therein, the term ε is introduced with a very small value to ensure numerical stability as the shear rate approaches zero.

(5) $$\eta_{\mathrm{app}}=\eta +\frac{\tau_{0}\left(|B|\right)\;\cdot \;\left[1-e^{-m_{p}\;\cdot \;\left|\dot{\gamma }\right|}\right]}{|\dot{\gamma }|\;+\;\varepsilon }.$$

In addition to the viscosity term η in the field-free state, Equation (5) also considers the formation of the magnetically induced yield stress and therefore replaces the tensor of the viscous shear stress K in Equation (3) with the modified term in Equation (6):

(6) $$K_{\mathrm{Bingh}}=\eta_{\mathrm{app}}\left(\nabla u+{\left(\nabla u\right)}^{T}\right).$$

### 4.2. Model for Describing the Motion of the Sphere

The displacement of the sphere is analyzed for a state of $\varphi =90^{\circ }$, when a chamber is crossing the center of the magnetic yoke. In this condition, the *y*-axis aligns with the central *r*-axis of chamber A, defined via the unit vector $e_{r}$, as displayed in Figure 1.

A tungsten carbide sphere with a diameter of $d_{\mathrm{sphere}}=9.15\;\mathrm{mm}$ is used as a balancing mass. This material was chosen because of its high mass density of 17.5 g cm^−3^ in relation to the MRF used. To guide the sphere laterally, the balancing chamber has a square cross-section with a side length of 10mm. The small clearance that allows for lateral movement of the balancing sphere is neglected in the following, and the motion of the sphere is restricted to a one-dimensional case, leaving displacement along the *r*-axis as the only degree of freedom. To describe the sphere’s position, its one-dimensional equation of motion can thus be formulated and solved in COMSOL Multiphysics. This is achieved by first determining the body and surface forces acting on the sphere before calculating the radial acceleration $\ddot{r}\;e_{r}$ using Newton’s second law. Therein $\sum F_{r}$ describes the sum of the forces acting on the balancing sphere in the *r*-direction.

(7) $$m\;\cdot \;\ddot{r}\;\cdot \;e_{r}=\sum F_{r}.$$

**Figure 10:**
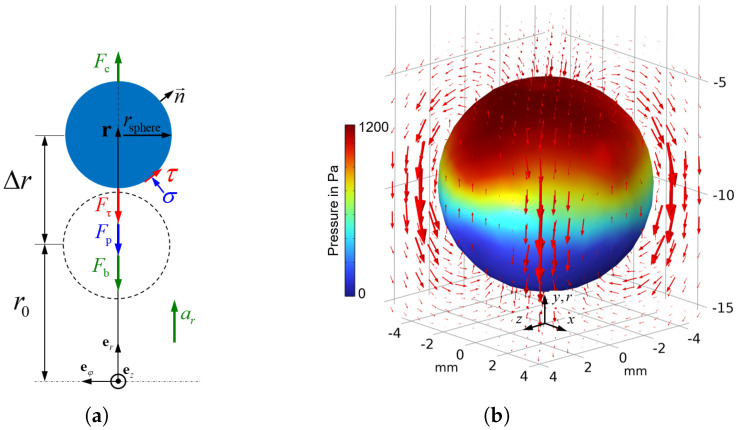
(**a**) Free body diagram of forces acting on the sphere. (**b**) Velocity field and pressures acting on the surface of the moving, balancing sphere.

The sphere’s position $r=r\;e_{r}$ can be obtained through double integration from its known initial position $r_{0}$, resulting in a corresponding change of mass distribution. If a free body diagram is illustrated for the sphere inside the chamber, as shown in Figure 10, the forces acting on the sphere can be described.

The rotational acceleration $a_{r}$, resulting from the angular velocity $\omega =\omega \;e_{\varphi }$ of the rotor and the distance *r* between the sphere’s center of mass and the axis of rotation, has a major impact on the sphere.

(8) $$a_{r}=r\;\cdot \;\omega^{2}\;e_{r}.$$

The product of this quantity and the mass of the balancing sphere $m_{\mathrm{sphere}}$ results in the centrifugal force $F_{c}=F_{c}\;e_{r}$.

(9) $$F_{c}=m_{\mathrm{sphere}}\;\cdot \;r\;\cdot \;\omega^{2}\;e_{r}.$$

Unlike the rotational acceleration $a_{r}$, which is always directed outward, the direction of the gravitational acceleration g depends on the angle φ. For the presented example, the rotational acceleration at an angular velocity of ω=50rad/s already exceeds the gravitational acceleration by a factor of 14, rendering the latter’s influence negligible during rotation.

Since the rotational acceleration acts not only on the balancing body but also on the surrounding MRF, the buoyancy force $F_{b}=F_{b}\;e_{r}$ must also be taken into account. This force acts against the centrifugal acceleration and is directed towards the axis of rotation. As described by Equation (10), wherein $V_{\mathrm{sphere}}$ corresponds to the volume of the balancing mass and $\rho_{\mathrm{MRF}}$ denotes the density of the MRF used, the buoyancy force is caused by the mass of the MRF displaced by the balancing body.

(10) $$F_{b}=-V_{\mathrm{sphere}}\;\cdot \;\rho_{\mathrm{MRF}}\;\cdot \;r\;\cdot \;\omega^{2}\;e_{r}.$$

Combining the terms for the buoyant force and the centrifugal force yields the outward force, which is determined by the difference in mass densities between the balancing body and the surrounding fluid, according to Equation (11):

(11) $$F_{c}+F_{b}=V_{\mathrm{sphere}}\;\cdot \;\left(\rho_{\mathrm{sphere}}-\rho_{\mathrm{MRF}}\right)\;\cdot \;r\;\cdot \;\omega^{2}\;e_{r}.$$

Equation (11) also shows the possibility of reversing the operating principle by inverting the density relationship. In the case that $\rho_{\mathrm{sphere}}<\rho_{\mathrm{MRF}}$, the direction of the resulting force is reversed, and the balancing body is driven toward the axis of rotation.

When considering the buoyant force, it should be noted that, depending on the chosen modeling approach, this force may also be included in the pressure forces described below. In this case, the effect of the centrifugal acceleration on the MRF must be considered in the Navier-Stokes-Equation. In the present case, the buoyant force is treated separately, as shown in Equation (11).

The forces acting on the surface of the balancing body are affected by the magnetically induced yield stress $\tau_{0}$ of the MRF and can be divided into a pressure-induced force $F_{p}=F_{p}\;e_{r}$ and a shear stress-induced force $F_{\tau }=F_{\tau }\;e_{r}$ depending on their cause. The pressure-induced component of the surface force acts in the direction of the surface normal vector n, which is defined in the chamber-fixed Cartesian coordinate system shown in Figure 10b where the *y*-axis is oriented in the *r*-direction. The component in the *r*-direction, relevant to the sphere’s movement, is obtained by integrating the pressure *p* over the sphere’s surface and taking the *y*-component $n_{y}$ of the surface normal $n={\left(n_{x},n_{y},n_{z}\right)}^{T}$ into account.

(12) $$F_{p}=\int_{A_{\mathrm{sphere}}}p\;\cdot \;n_{y}\;\;dA.$$

The surface force $F_{\tau }$ resulting from shear stress is obtained by integrating the shear stress τ over the sphere’s surface. For the movement of the sphere, the *r*-component of this force $F_{\tau }$ is decisive, which is calculated in the same way as the pressure force by taking into account the corresponding components of the surface vector according to Equation (13). To calculate the shear stress acting on the surface, the Bingham-Papanastasiou-Approach already used for fluid behavior is applied.

(13) $$F_{\tau }=\int_{A_{\mathrm{sphere}}}\tau_{0}\left(|B|\right)\;\cdot \;\left[1-e^{-m\;\cdot \;|\dot{\gamma }|}\right]\;\cdot \;\sqrt{n_{x}^{2}+n_{z}^{2}}\;\cdot \;\mathrm{sgn}\left(\dot{\gamma }\right)\;\;dA.$$

If only the *r*-component of the forces described are used, it is possible to simplify the representation of the equilibrium of forces in Equation (14).

(14) $$m\;\cdot \;\ddot{r}=F_{c}+F_{b}-F_{\tau }-F_{p}.$$

Rearranging the equation then provides the required acceleration of the balancing body:

(15) $$\ddot{r}=\frac{F_{c}+F_{b}-F_{\tau }-F_{p}}{m_{\mathrm{sphere}}}.$$

By integrating the acceleration and taking into account the initial value ${\dot{r}}_{0}$, the velocity $\dot{r}$ of the sphere can be determined according to Equation (16) and its displacement Δr according to Equation (17). The therm $r_{0}$ represents the initial distance between the sphere and the axis of rotation, as depicted in Figure 10a.

(16) $$\dot{r}=\int \ddot{r}\;dt+{\dot{r}}_{0}.$$

(17) $$\Delta r=\int \dot{r}\;dt=r-r_{0}.$$

This equation of motion for the balancing body is implemented and solved in COMSOL Multiphysics. Regarding the surface forces $F_{p}$ and $F_{\tau }$, it should also be noted that they are a consequence of the sphere’s motion. As the sphere moves toward the outer region of the chamber, a corresponding counter-flow of the MRF must occur. Due to the square cross-section of the chamber, it is not possible to model the flow phenomenon in two dimensions. This means that the three-dimensional Navier–Stokes-Equation must be solved. To couple the sphere’s motion with the flow field, the deformed physics feature is used. The displacement determined from the equation of motion is specified at the boundary of the mesh that defines the balancing sphere. This deforms the fluid mesh and enforces a counterflow in the surrounding MRF, as depicted in Figure 10b.

The deformable mesh used for the flow simulation only models the interior of the chamber, specifically the MRF and the balancing sphere. It consists of approximately 16,000 elements, including 15,384 tetrahedrons and 424 pyramids. In order to calculate the magnetic field, the mesh was expanded to include the remaining elements of the chamber, the electromagnet, and the surrounding space. For efficiency, this air space was meshed relatively coarsely and extended at the boundaries using infinite element domains. This results in a total number of approximately 32,000 elements for the magnetic simulation.

**Figure 11:**
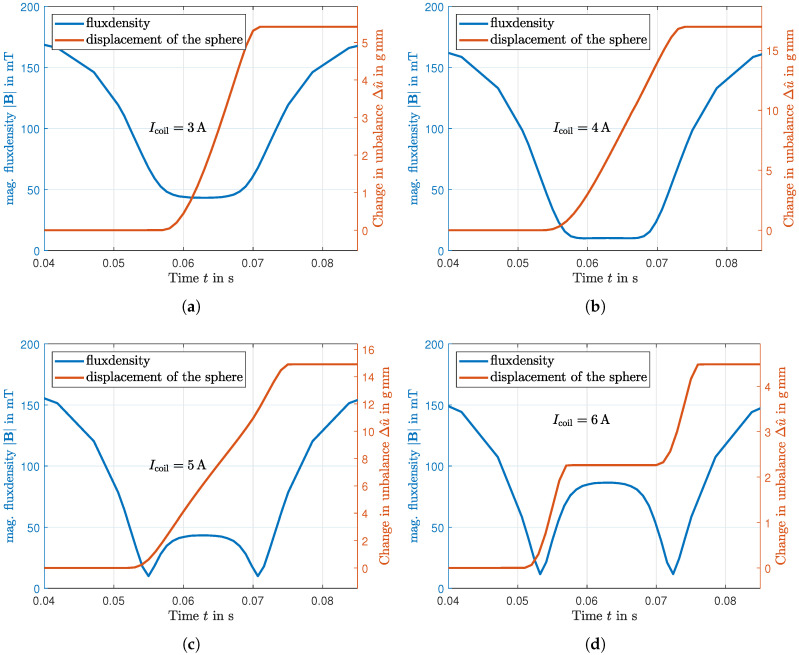
Absolute value of the magnetic flux density over time, averaged over the fluid volume of the chamber and the resulting displacement of the balancing sphere over time. (**a**) For a current of $I_{\mathrm{coil}}=3\;A$, (**b**) for a current of $I_{\mathrm{coil}}=4\;A$, (**c**) for a current of $I_{\mathrm{coil}}=5\;A$, (**d**) for a current of $I_{\mathrm{coil}}=6\;A$.

Figure 11a–d illustrate the results of the chamber passing through the air gap when the electromagnet is activated at coil currents of $I_{\mathrm{coil}}=3-6\;A$. Each representation shows the absolute value of the magnetic flux density |B| experienced by the MRF surrounding the sphere and the resulting change in the amplitude of the unbalance $\Delta \hat{u}$, which corresponds to the displacement of the sphere. In Figure 11a, at a coil current of 3A, the compensation of the magnetic field is just sufficient to induce a small displacement of the sphere. Figure 11b represents the best compensation in the center of the air gap, where a current of 4A provides maximum field displacement. This configuration results in a smooth and continuous motion of the sphere across the gap. When increasing the current to 5A, as depicted in Figure 11c, an overcompensation of the field in the center is obtained. However, this creates a wider region of compensation towards the edges of the gap, allowing the sphere to move over a longer period of time, but with a lower average velocity, resulting in a smaller variation of unbalance $\Delta \hat{u}$. Finally, Figure 11d demonstrates the effect of significant overcompensation with a current of 6A. This condition allows the sphere to move only when entering and leaving the air gap, leading to an intermittent stop in the center where the opposing magnetic field increases the yield stress $\tau_{0}$, thereby halting the sphere temporarily. Due to this, only a slight shift of the balancing mass is caused, which means that this case should not be chosen for efficiency reasons.

## 5. Setup for Experimental Analysis of the Balancing Process and Experimental Parameterization

Section 5 presents the system for the experimental analysis and the required parameterization for the balancing control. After the parameterization procedure is described, the results are presented and compared with the simulation data in order to validate the models created.

### 5.1. Setup of the Test Stand and Description of the Unbalance Measurement

**Figure 12:**
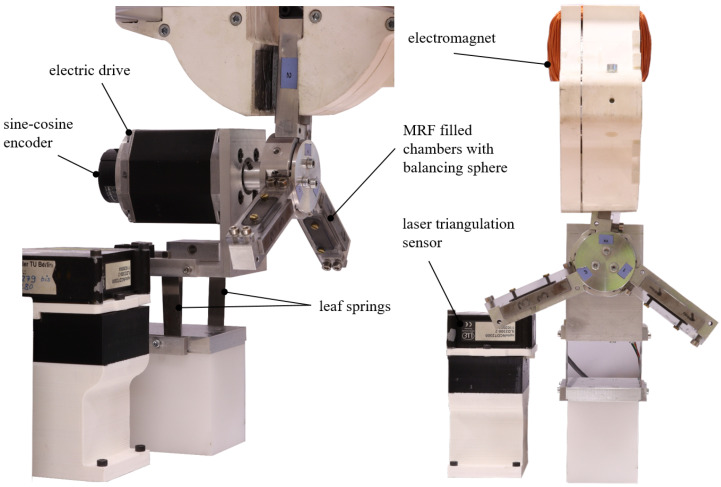
Setup of the test stand used for parameterization and execution of the balancing runs with the mounted balancing head.

For the experimental analysis, the test rig shown in Figure 12 is used. This system is based on the approach already presented in [18], but has been revised to allow measurements at higher angular velocities and to enable the reverse movement of the balancing spheres for further balancing processes. It consists of the electric drive, a measuring system for model-based identification of the unbalance, and the rotor in which the online balancing system is embedded. A real-time system from dSPACE is used for control of the test stand and to record the measurement data.

The drive system consists of a BG 65×25 brushless DC machine from Dunkermotoren GmbH l(Bonndorf, Germany), which is driven by a CDF3000 inverter from KEBA Industrial Automation Germany GmbH (Lahnau, Germany). The position φ of the rotor is recorded by an ERO 1480 sine-cosine encoder from DR. JOHANNES HEIDENHAIN GmbH (Traunreut, Germany), mounted on the drive shaft and evaluated by the real-time system MicroLabBox from dSPACE GmbH (Paderborn, Germany) in terms of position and angular velocity. This information is used for speed and position control, the unbalance measurement, and to control the balancing system via the electromagnet.

### 5.2. Experimental Parameterization of the Displacement of the Sphere

**Figure 13:**
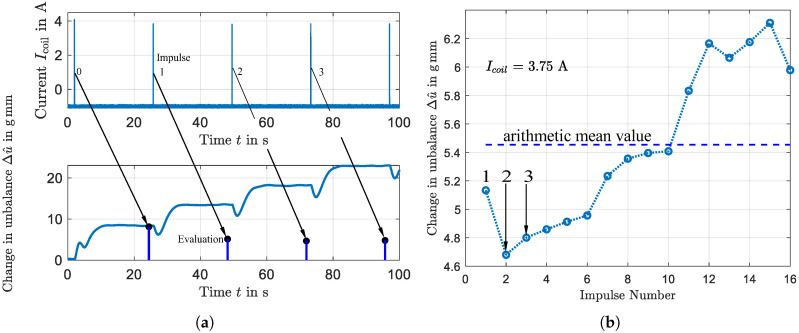
(**a**) Time-domain representation of the parameterization process. Top: Actuation signal showing current pulses $I_{\mathrm{coil}}$ applied to the coil. Bottom: Course of the unbalance and evaluation points. (**b**) Corresponding analysis of unbalance change $\Delta \hat{u}$ per impulse for a current $I_{\mathrm{coil}}=3.75A$. The horizontal line represents the average change over 16 impulses.

In order to parameterize the balancing algorithm used in Section 6, the effect of the different current amplitudes must be identified. For this purpose, parameterization runs with chamber B were carried out in increments of ΔIcoil=0.125 A respectively ΔIcoil=0.25 A after peak displacement. Figure 13a illustrates a segment of such a parameterization sequence for an impulse amplitude of Icoil=3.75 A. The top subplot displays the actuation signal, which consists of periodic current pulses applied to the electromagnetic coil. Each pulse is only active when chamber A has just left the gap, and only until chamber B has passed through completely. To minimize boundary effects that can occur when the balancing body is touching the inner chamber wall, a single reference pulse with an amplitude of Icoil=4 A is applied before each parameterization begins. Subsequent pulses use the desired amplitude for parameterization with Icoil=3.75 A in the case presented.

The bottom subplot of Figure 13a shows the corresponding system response, displaying the change in the rotor unbalance, $\Delta \hat{u}$ over time. The initial unbalance is zero. Following each current pulse shown in the top plot, the unbalance exhibits a distinct stepwise increase. The numbered vertical lines mark the magnitude of the unbalance change achieved after each pulse of actuation. To further analyze the precision and repeatability of the process, the effect of each individual impulse was quantified. Figure 13b presents the change in unbalance $\Delta \hat{u}$, achieved per impulse for a series of 16 consecutive actuations. The data shown represents the complete parameterization whose first four pulses are shown in Figure 13a and show that although each impulse successfully increases the unbalance, there are certain differences in the amount of the unbalance change. The horizontal line represents the average unbalance change across all impulses. A possible explanation for the visible increase could be the growing distance between the center of the sphere and the axis of rotation $r_{\mathrm{loc}}$ and its effect on the force $F_{\omega }$ shown in Equation (9).

### 5.3. Results of the Parameterization Process and Comparison with the Simulation Outputs

**Figure 14:**
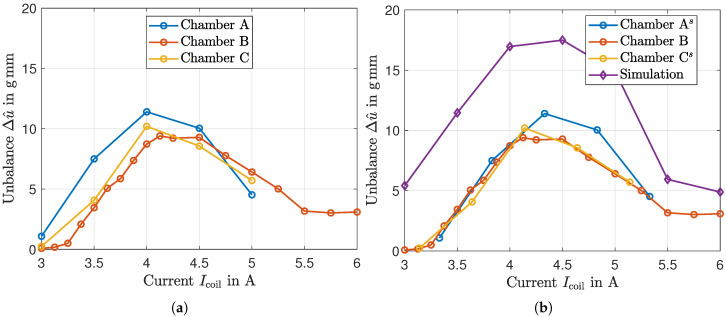
Displacement of the balancing ball when passing through the air gap with activated electromagnet as a dependence of the coil current. The plot compares experimental data with results from the simulation. (**a**) Recorded measurement data of three chambers, (**b**) measurement data of chamber A and C shifted (superscript *s*) according to offset determined in the magnetic parameterization (see Figure 5b).

The parameterization process for chamber B was repeated for a range of current amplitudes, $I_{\mathrm{coil}}\;=\;3\;-\;6\;A$, to fully characterize the system response. The average unbalance change per impulse was calculated for each current level. Figure 14a summarizes these findings and compares the results of chamber B with the results of the other two chambers, A and C. The experimentally obtained curve of chamber B shows a strong nonlinear dependence between the applied current and the resulting change in unbalance. No change in unbalance is measured at currents below 3 A. As the current increases, the displacement per impulse grows, reaching a peak effectiveness at approximately 4.25 A. Beyond this point, the change achieved decreases again. This fundamental behavior was already observed in the simulation results in Figure 11. There are certain deviations between the curves of the three chambers examined. However, if the offset of the compensation current shown in Figure 5b is taken into account by shifting the characteristic curves of chambers A and B by the determined values $\Delta I_{A}$ and $\Delta I_{C}$, good correlation is achieved, which is illustrated in Figure 14b.

Figure 14b also includes results from a numerical simulation. From a quality point of view, the simulation almost predicts the trend of the applied current but significantly overestimates the magnitude of the displacement. While the current-dependent variation in unbalance between simulation and measurement corresponds well, the simulation shows a significantly greater change, which approximately represents an offset. In addition to the deviation in the underlying magnetic flux density (Figure 7a), the simplified coupling of the simulation could be a possible explanation. In the presented work, only the mean value of the magnetic flux density is determined and applied uniformly to the entire fluid volume. However, due to the complex geometry, different local values of the magnetic flux density, and thus the yield point, are to be expected. These local differences are not taken into account by the modeling approach in Section 4.1 and can affect the forces on which the equation of motion is based, possibly causing the simulation to overestimate the investigated effect.

## 6. Control Algorithm for Time-Efficient Unbalance Correction

**Figure 15:**
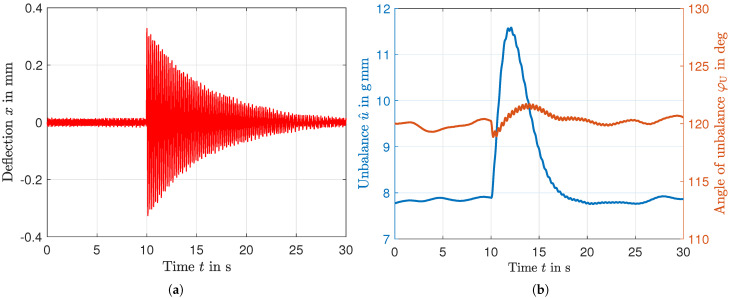
Reaction of the unbalance measurement to a disturbance impulse at t=10 s. (**a**) Deflection detected by the laser distance sensor, (**b**) determined values for magnitude and phase of the unbalance measurement by phase-sensitive demodulation [18].

Section 6 describes the algorithm used for time-efficient balancing, which is adapted to the slow decaying behavior of the unbalance measurement. To minimize the effects of unbalance variations occurring during operation, corrections must be made as soon as possible. The time interval required for these adjustments depends on two main factors: the time needed to identify the change in unbalance and the time needed to complete the correction. The method of unbalanced measurements, described in more detail in [18], is based on deflection and exploits the low stiffness of the leaf springs in the x-direction. A disadvantage of this method is the long settling time that occurs, as demonstrated in Figure 15, where deflection and the measured unbalance state are visualized over time. At time t=10s an external disturbance was applied by tapping the support structure. Although this disturbance has no effect on the rotor’s unbalance, it does affect the measurement. As detailed in [18], the evaluation only demodulates the rotor frequency, while the system, disturbed by the impulse, oscillates at its natural frequency. Consequently, the amplitude of the deflection in Figure 15b, only decays slowly, whereas the measured values for phase and amplitude have already approached their initial values after about 10 s.

**Figure 16:**
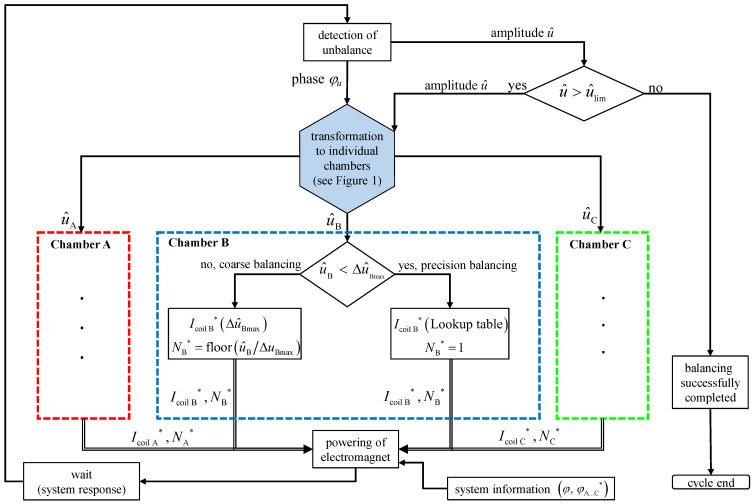
Algorithm used for online balancing. The sequence for chambers A and C corresponds to the path in the loop shown for chamber B.

Since every manipulation during the balancing process requires a pulse, the algorithm presented below aims to minimize the number of necessary interventions. This balancing algorithm, depicted in Figure 16 operates in an iterative loop. Initially, it determines the amplitude and phase of the unbalance. If the amplitude exceeds a predefined limit, the total unbalance state is split into individual correction targets for the three balancing chambers. These targets are processed in parallel but in coordination. The sequence is shown for chamber B in Figure 16 as an example. First, a case distinction is used to determine whether the correction can be carried out in one step. This is done by comparing the required correction ${\hat{u}}_{B}$ in the direction of chamber B with the maximum change determined from the parameterization ${\hat{u}}_{\max}$. If the required correction exceeds the change possible in one step $\hat{u}>\Delta {\hat{u}}_{\max}$, the number of pulses required is determined by division and subsequent rounding down $N=\mathrm{floor}\left(\hat{u}/\Delta {\hat{u}}_{\max}\right)$. If the required value can be compensated with one pulse $\hat{u}<\Delta {\hat{u}}_{\max}$, the current required for this single step is determined by interpolating the parameterization data. According to this scheme, the current amplitude and number of impulses from all three chambers are determined and combined with respect to the actuation angle of each chamber. Next, the electromagnet is instantly activated. After the actuation of the electromagnet, the algorithm pauses to allow the system to settle before carrying out a new measurement and repeating the loop. This process continues until the unbalance is successfully reduced below the target threshold $\hat{u}<{\hat{u}}_{\lim}$.

## 7. Experimental Validation of the Proposed Balancing System

**Figure 17:**
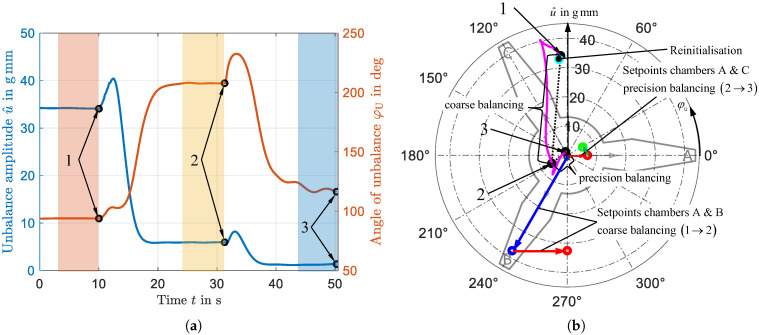

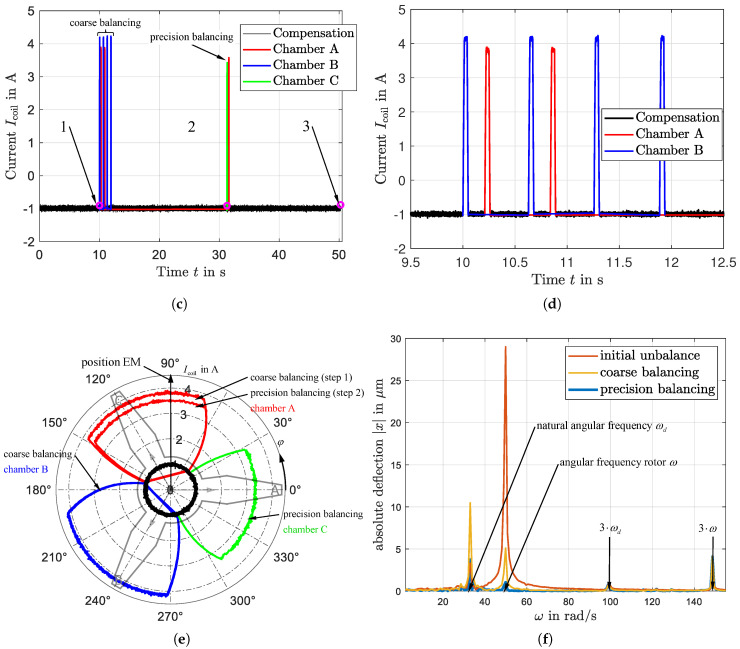
Results of the representative balancing run were examined. (**a**) Time curve of amplitude and phase of the unbalance measurement, (**b**) polar diagram of the balancing and reinitialization process, (**c**) sequence of the currents applied to the electromagnet, (**d**) magnified representation of the current pulses during the first balancing phase, (**e**) polar diagram of the currents applied to the electromagnet activating the chambers, (**f**) deflection displayed in the frequency domain; in particular, the first harmonic, which is excited by the unbalance, is significantly reduced.

In Section 7, the performance of the system is presented on the basis of an exemplary balancing run. The test rig introduced in Section 5 and the algorithm presented in Section 6 are used for the investigation. As an initial state, an unbalance is applied by attaching weights to the rotor and accelerating the system to an angular velocity of ω=50rad/s. After activation of the balancing algorithm, the unbalance is then automatically corrected. Figure 17a shows the course of the experiment, demonstrating the evolution of the unbalance amplitude $\hat{u}$ and phase $\varphi_{u}$ over time. At t = 0 s, the system is in a steady state with an initial unbalance of $\hat{u}=34.5\;g\;\mathrm{mm}$ at a phase angle of $\varphi_{u}=94^{\circ }$. Due to the phase of the unbalance, a displacement of the balancing spheres in chambers A and B is required for correction. Activating the algorithm at t = 10 s initiates this displacement. Due to the magnitude of the unbalance, a two-stage process involving coarse and precise balancing is required; see the blue dashed frame in Figure 16. The designated setpoints for the two steps, coarse and precision balancing, are shown in Figure 17b for the chambers involved. As can be seen from the current profile in Figure 17c, several pulses are visible immediately after activation. Figure 17d provides a magnified view of these currents during the coarse balancing phase. Initially, two pairs of pulses are visible, corresponding to the values for the maximum displacement of the sphere in each chamber, determined during parameterization. Chamber A requires a current of 3.8 A, while Chamber B utilizes 4.13 A. The difference between these two setpoint values results from the shift in the characteristic curves between chambers A and B, as detailed in Table 2. Due to the unbalance’s phase angle $\varphi_{u}$, a larger displacement is required in chamber B, prompting the application of two additional pulses; see Figure 17d. As shown in Figure 17a, the pulses initially cause a slight increase in the measured unbalance before it decreases to approximately $\hat{u}=6\;g\;\mathrm{mm}$. Once the unbalance measurement reaches a steady state, the algorithm is triggered again. Due to the small remaining unbalance, it now operates in precision mode, requiring only one activation per chamber. Because of the slight overshoot during the coarse balancing step, chambers A and C require compensation in the second precision balancing step. The current required for complete compensation is determined using the experimentally parameterized look-up table and is illustrated in a polar plot in Figure 17e, providing a spatial assignment to the respective chambers. Taking chamber A as an example, the two different amounts of current for the first coarse (step 1) and precision (step 2) balancing are clearly visible. After the system settles, a final residual unbalance of $\hat{u}=1.2\;g\;\mathrm{mm}$ at a phase angle of $\varphi_{u}=117^{\circ }$ remains.

The process can also be tracked by the polar plot in Figure 17b, where the alignment of the three chambers is visible in the background. The continuously measured value (magenta) follows a curved path caused by the feedback of the balancing impulses. In contrast, the actual unbalance state of the rotor is represented by the black dots, which mark the steady-state values after each step.

Additionally, the cyan dot in Figure 17b indicates the measured unbalance after the reinitialization of the three chambers. For this purpose, the chambers were positioned one after the other in the air gap of the magnetic yoke and energized with a current varying around the compensation value. After completing the reinitialization procedure, the resulting value of $\hat{u}=32.8\;g\;\mathrm{mm}$ at $\varphi_{u}=95^{\circ }$ closely matches the initial unbalance, confirming that the balancing spheres had been successfully returned to their starting positions.

Figure 17f shows the result of the balancing process in the frequency range. The time spans utilized to determine the amplitude spectrum cover a time span of seven seconds each, which are highlighted in a corresponding color in Figure 17a. In particular, the first harmonic of the amplitude spectrum of the recorded deflection, which is excited by the unbalance, is significantly reduced.

## 8. Discussion and Conclusions

This study successfully demonstrated the design, implementation, and experimental validation of an online rotor balancing system utilizing the movement of MRF embedded balancing spheres. A main advantage of the system, maintaining the corrected state without a continuous energy supply, results from the hybrid magnetic design. Using permanent magnets for passive locking and an electromagnet for active correction, the system offers inherent stability and energy efficiency.

A main focus of this work is the model-based approach combined with experimental investigations, which provided key insights into the system’s behavior. To gain a deep understanding of the internal dynamics, a comprehensive three-dimensional multiphysics model was developed. By incorporating the non-Newtonian fluid behavior using the Bingham-Papanastasiou approach, the model successfully predicted the qualitative and, to a large extent, quantitative behavior of the balancing sphere’s displacement as a function of the coil current, and correctly determined optimal current values for maximum effectiveness. However, the simulation overestimated the magnitude of the displacement. This discrepancy is due to simplifications in the modeling, in which an average magnetic flux density was applied uniformly to the entire MRF volume, whereas in reality, local variations in the yield stress may occur. This deviation suggests that future modeling efforts should incorporate a more detailed, spatially resolved field distribution to improve quantitative accuracy. Similarly, the assumption of a centered sphere may not reflect reality. If the balancing sphere comes into contact with one of the chamber walls during operation, additional boundary effects that have not been taken into account could occur. Nevertheless, the developed simulation provides a solid foundation for the development of further systems.

For automated online balancing, a time-efficient control algorithm was developed. Its proposed two-stage approach, separating coarse and fine adjustments, is key to the system’s ability to perform rapid and precise balancing with a minimum number of active interventions. Through experimental parameterization of the magnetic circuits as well as the displacement of the spheres, deviations between the individual chambers could be identified and compensated for by the algorithm. The experimental results of a representative balancing process show the system’s effectiveness in reducing an initial unbalance of approximately 34.5 g mm to a value of just 1.2 g mm. This reduction of 96% is achieved with just two active interventions using the developed algorithm, thus proving the performance of the online balancing system.

Based on the findings in this contribution, several opportunities for future research can be identified. First, refining the multiphysics model to incorporate non-uniform field effects could increase the accuracy of the simulations and support the design of future hardware. Second, exploring alternative or supplementary unbalance measurement techniques with faster responses could significantly reduce the balancing cycle time. Third, enhancing the control algorithm to be more adaptive, perhaps by using a model-based observer to predict the final unbalance state without waiting for the system to settle fully, could further improve time efficiency. Finally, the investigation of potential methods to minimize variance between chambers would serve to simplify control and improve the robustness of the system. Despite some potential for improvement, this work provides a strong foundation and a successful concept for a stable, effective, and energy-efficient online rotor balancing system.

## Figures and Tables

**Table 1:** Properties and characteristic of the configuration analyzed in this work.

Parameter	Symbol	Value	Unit
Coil windings	*N*	400	-
Dimensions PM	-	10×10×2	${\mathrm{mm}}^{3}$
Magnetization PM		N45	
max. displacement	$\max.\left(\Delta r\right)$	22.65	mm
max. unbalance correction	$\max.(\Delta \hat{u})$	121.6	g mm
Radius sphere	$r_{\mathrm{sphere}}$	4.575	mm
Mass sphere	$m_{\mathrm{sphere}}$	7.02	g
Mass density sphere	$\rho_{\mathrm{sphere}}$	17.5	g/${\mathrm{cm}}^{3}$
Angular velocity	ω	50	rad/s

**Table 2:** Comparison of the coil currents required for field displacement of the three permanent magnetic circuits used.

Chamber	Compensation Current	Offset
Chamber A	$I_{\mathrm{compA}}=3.67\;$ A	$\Delta I_{A}=0.33\;$ A
Chamber B	$I_{\mathrm{compB}}=4.00\;$ A	$\Delta I_{B}=0.00\;$ A
Chamber C	$I_{\mathrm{compC}}=3.86\;$ A	$\Delta I_{C}=0.14\;$ A

**Table 3:** Selected technical data of the MRF Basonetic 5030 used [22].

Particle volume concentration	$C_{V}=47\%$
Density	$\rho_{\mathrm{MRF}}=4.12\;g\;{cm}^{-3}$
Relative permeability at |B|≈0	$\mu_{r}=\mu /\mu_{0}=7.96$
Yield stress at |B|=0.1T	$\tau_{0}=2340\mathrm{Pa}$
Dynamic viscosity at ϑ=20 °C	η=0.4Pas
Bingham-Papanastasiou exponent	$m_{p}=10$

## Data Availability

The raw data supporting the conclusions of this article will be made available by the authors on request.

## Abbreviations

The following abbreviations are used in this manuscript:

MRF	Magnetorheological Fluid
EM	Electromagnet
PM	Permanentmagnet
